# Dataset reporting the perceiver identification rates of basic emotions expressed by male, female and ambiguous gendered walkers in full-light, point-light and synthetically modelled point-light walkers

**DOI:** 10.1016/j.dib.2017.11.005

**Published:** 2017-11-07

**Authors:** Shaun Halovic, Christian Kroos

**Affiliations:** MARCS Institute, Western Sydney University, Australia

## Abstract

This data set describes the experimental data collected and reported in the research article “Walking my way? Walker gender and display format confounds the perception of specific emotions” (Halovic and Kroos, in press) [1]. The data set represent perceiver identification rates for different emotions (happiness, sadness, anger, fear and neutral), as displayed by full-light, point-light and synthetic point-light walkers. The perceiver identification scores have been transformed into H*t* rates, which represent proportions/percentages of correct identifications above what would be expected by chance. This data set also provides H*t* rates separately for male, female and ambiguously gendered walkers.

**Specifications Table** *[please fill in right-hand column of the table below]*

**Table:** 

Subject area	*Psychology*
More specific subject area	*Psychophysics*
Type of data	*SPSS file*
How data was acquired	*Perceiver identification rates were collected over three experiments as they viewed walkers displaying different basic emotions*
Data format	*Converted to Ht scores (see**[1]**for more details)*
Experimental factors	*Male and female walkers displayed four basic emotions (happy, sad, anger and fear), and a neutral comparison, through their walking movements. These walkers were shown to perceivers in full-light and point-light display formats. Synthetically modelled point-light walkers were also shown to perceivers, which were normalized for walker structure and form and deleted any idiosyncratic movements that may have confused emotion perception. Synthetically modelled walkers were constructed separately from male and female kinematic data and then jointly to create gender ambiguous walkers.*
Experimental features	*Perceivers viewed walker stimuli and attempted to identify which basic emotion that walker was expressing. Perceiver responses were recorded using experiment-control software Alvin**[2]*.
Data source location	*Sydney, Australia*
Data accessibility	*Data submitted with this article*

**Value of the data**

• Data expresses perceiver identification rates as H*t* rates, which control for repeated stimulus presentation and minimize observer response bias, inaccurate calculations of chance and the overestimation of perceiver performance.
• Synthetically modelled walker stimuli control for differences in walker form and isolates the kinematics underlying each emotional expression for male, female and gender ambiguous walkers.
• The entanglement of emotion perception and emotion expression that have plagued past research have been untangled in our synthetic walker stimuli. Future psychophysical studies can thus manipulate perceptual variables with the understanding that any differences found in perceiver identification rates, relative to our data, are due to the effect of their experimental manipulations.
• Other psychophysical researchers can contact the primary author for use of our synthetically modelled point-light walker stimuli.

## 1 Data

The data set are perceiver identification rates for four basic emotions (happiness, sadness, anger and fear) with a neutral comparison, as displayed by full-light, point-light and synthetic point-light walkers. The scores have been transformed into H*t* rates [1] and presented as percentages. This data set also provides H*t* rates separately for male, female and ambiguously gendered walkers.

## 2 Experimental design, materials and methods

This article reports on the data collected over three perceptual experiments. 36 professional actors were recorded with a video camera and a Vicon motion capture system, as they expressed four basic emotions (happiness, sadness, anger and fear) and a neutral comparison through their walking movements. Full-light walker stimuli were created from the video recordings and shown to perceivers in experiment 1. Point-light walker stimuli were created in Matlab (The MathWorks, inc) from the Vicon motion data recordings and shown to perceivers in experiment 2. Synthetically modelled walker stimuli were created using an extension of Troje's techniques [3], and shown to perceivers in experiment 3. The stimuli in each experiment comprised male and female walkers, and in experiment 3, additional gender ambiguous walkers.

The emotion displaying walker stimuli were presented to perceivers through a computer running experiment-control software Alvin [2]. The presentation order of all stimuli in each experiment were counter-balanced with an inter-stimulus interval of 2 s. Perceiver participants were required to click an on-screen button that corresponded to the emotion that they thought the walking stimulus was displaying.

The raw frequency scores were converted to H*u* scores [4] and then further to H*t* scores [1]. The H*t* scores presented in this dataset represent the congruence between the displayed emotion and the perceived emotion.
