# Supplementary material for: Dataset reporting the perceiver identification rates of basic emotions expressed by male, female and ambiguous gendered walkers in full-light, point-light and synthetically modelled point-light walkers
Source: Data Brief. 2017 Nov 7;15:1000–2. doi: 10.1016/j.dib.2017.11.005 (PMC5684099; doi:10.1016/j.dib.2017.11.005)
Supplement: Supplementary file 1 — Supplementary material [file mmc1.pdf]

Journal of Epidemiology and GLOBAL HEALTH

ELSEVIER

# CONFLICT OF INTEREST DECLARATION AND AUTHOR AGREEMENT FORM

**It is important that you return this form upon submission. We will not publish your article without completion and return of this form.**

**Title of Paper:** Dataset reporting the perceiver identification rates of basic emotions expressed by male, female and ambiguous gendered walkers in full-light, point-light and synthetically modelled point-light walkers

Please tick one of the following boxes:

☑ We have no conflict of interest to declare.

☐ We have a competing interest to declare (please fill in box below):

This statement is to certify that all Authors have seen and approved the manuscript being submitted. We warrant that the article is the Authors' original work. We warrant that the article has not received prior publication and is not under consideration for publication elsewhere. On behalf of all Co-Authors, the corresponding Author shall bear full responsibility for the submission.

This research has not been submitted for publication nor has it been published in whole or in part elsewhere. We attest to the fact that all Authors listed on the title page have contributed significantly to the work, have read the manuscript, attest to the validity and legitimacy of the data and its interpretation, and agree to its submission to the *Journal of Epidemiology and Global Health.*

All authors agree that author list is correct in its content and order and that no modification to the author list can be made without the formal approval of the Editor-in-Chief, and all authors accept that the Editor-in-Chief's decisions over acceptance or rejection or in the event of any breach of the Principles of Ethical Publishing in the *Journal of Epidemiology and Global Health* being discovered of retraction are final.

No additional authors will be added post submission, unless editors receive agreement from all authors and detailed information is supplied as to why the author list should be amended.

| **Author Signature** | **Print Name** |
|---|---|
| [signature] | Shaun Halovic |
| | |
| | |
| | |

☑ Please check this box if you are submitting this on behalf of all authors.
